# Leucine and Protein Metabolism in Obese Zucker Rats

**DOI:** 10.1371/journal.pone.0059443

**Published:** 2013-03-20

**Authors:** Pengxiang She, Kristine C. Olson, Yoshihiro Kadota, Ayami Inukai, Yoshiharu Shimomura, Charles L. Hoppel, Sean H. Adams, Yasuko Kawamata, Hideki Matsumoto, Ryosei Sakai, Charles H. Lang, Christopher J. Lynch

**Affiliations:** 1 Department of Cellular and Molecular Physiology, the Pennsylvania State University College of Medicine, Hershey, Pennsylvania, United States of America; 2 Department of Applied Molecular Biosciences, Graduate School of Bioagricultural Sciences, Nagoya University, Nagoya, Japan; 3 Department of Pharmacology, Case Western Reserve University, Cleveland, Ohio, United States of America; 4 Obesity and Metabolism Research Unit, USDA-Agricultural Research Service Western Human Nutrition Research Center, and Department of Nutrition, University of California Davis, Davis, California, United States of America; 5 Institute for Innovation, Ajinomoto. Co., Inc., Kawasaki, Japan; National Institute of Agronomic Research, France

## Abstract

Branched-chain amino acids (BCAAs) are circulating nutrient signals for protein accretion, however, they increase in obesity and elevations appear to be prognostic of diabetes. To understand the mechanisms whereby obesity affects BCAAs and protein metabolism, we employed metabolomics and measured rates of [1-^14^C]-leucine metabolism, tissue-specific protein synthesis and branched-chain keto-acid (BCKA) dehydrogenase complex (BCKDC) activities. Male obese Zucker rats (11-weeks old) had increased body weight (BW, 53%), liver (107%) and fat (∼300%), but lower plantaris and gastrocnemius masses (−21–24%). Plasma BCAAs and BCKAs were elevated 45–69% and ∼100%, respectively, in obese rats. Processes facilitating these rises appeared to include increased dietary intake (23%), leucine (Leu) turnover and proteolysis [35% per g fat free mass (FFM), urinary markers of proteolysis: 3-methylhistidine (183%) and 4-hydroxyproline (766%)] and decreased BCKDC per g kidney, heart, gastrocnemius and liver (−47–66%). A process disposing of circulating BCAAs, protein synthesis, was increased 23–29% by obesity in whole-body (FFM corrected), gastrocnemius and liver. Despite the observed decreases in BCKDC activities per gm tissue, rates of whole-body Leu oxidation in obese rats were 22% and 59% higher normalized to BW and FFM, respectively. Consistently, urinary concentrations of eight BCAA catabolism-derived acylcarnitines were also elevated. The unexpected increase in BCAA oxidation may be due to a substrate effect in liver. Supporting this idea, BCKAs were elevated more in liver (193–418%) than plasma or muscle, and per g losses of hepatic BCKDC activities were completely offset by increased liver mass, in contrast to other tissues. In summary, our results indicate that plasma BCKAs may represent a more sensitive metabolic signature for obesity than BCAAs. Processes supporting elevated BCAA]BCKAs in the obese Zucker rat include increased dietary intake, Leu and protein turnover along with impaired BCKDC activity. Elevated BCAAs/BCKAs may contribute to observed elevations in protein synthesis and BCAA oxidation.

## Introduction

Branched chain amino acids [BCAAs, including leucine (Leu), valine (Val) and isoleucine (Ile)] are important nutrient signals increasing insulin secretion in islet beta cells and mammalian Target of Rapamycin (mTOR) signaling in most tissues. In addition, they regulate satiety and affect glucose metabolism through peripheral and central mechanisms [1], [2], [3], [4], [5], [6]. In obesity, elevated concentrations of plasma BCAAs have been frequently reported in humans and rodent models starting in the late 1960 s [7], [8]. Elevations in BCAAs and related metabolites have been described as a “metabolic signature” for obesity, insulin resistance and glucose intolerance [9]. Felig and coworkers speculated that the hyperaminoacidemia, as opposed to hyperglycemia, sustained insulin resistance and thereby promoting hyperinsulinemia in obesity [8]. Adding to this, Um et al [10], showed that hyperaminoacidemia increased mTOR activity through an insulin-independent and direct effect on muscle that additionally promoted insulin resistance. The importance of the link between elevated BCAAs and obesity has been buoyed by analysis of data from the Framingham study showing that elevated plasma concentrations of BCAAs, tyrosine and phenylalanine (Phe) were prognostic of type-2 diabetes (T2D), a disease in which BCAAs are also generally elevated [11]. Although it has been proposed that BCAAs drive some of the insulin resistance phenotype through activation of mTOR under high fat feeding conditions [9] or in human obesity [8], this remains controversial. Other studies have indicated beneficial metabolic effects of BCAA or BCAA-rich diets, and associations between insulin action and BCAA-specific activation of mTOR are not entirely consistent [12], for review see [13]. This raises the possibility that elevations in circulating BCAA reflect insulin resistance and are not causative.

To better understand how BCAAs associate with the pathogenesis of obesity and diabetes, it is necessary to determine the mechanisms underlying their elevation. Plasma BCAA concentrations result from the difference between their rates of appearance and disappearance. These are due on the one hand to dietary intake and tissue proteolysis, and counterbalanced by protein synthesis and BCAA catabolism. It is possible that elevated plasma BCAA concentrations in obesity and diabetes are secondary to decreased protein synthesis and/or elevated protein degradation resulting from insulin resistance as originally speculated by Felig and coworkers [8] or, depending on the model or stage of obesity development, hyperphagia and concurrent increased amino acid intake might play a role. Indeed, using [1-^13^C]-Leu and gas-chromatography mass spectrometry (GC-MS), elevated protein turnover and proteolysis in obese humans has been reported [14], [15], [16], [17], [18], [19]. However this too has not always been observed [20], [21] and seems inconsistent with the findings that aminoacidemia in lean animals and in obesity activates mTOR kinase activity. In lean animals, such signaling by BCAAs increases protein synthesis and inhibits muscle protein degradation as measured by changes in 3-methylhistidine (3-MeHis) [10], [22], [23]. However skeletal muscle mass is reduced in obese Zucker rats consistent with increased 3-MeHis [24], [25], [26].

Given the highly regulated nature of BCAA catabolism, diminished or incomplete BCAA catabolism could also contribute to elevations in BCAAs. Recent metabolomic, proteomic and genomic studies have suggested altered BCAA metabolism as a potential etiology with some studies focusing on adipose tissue [9], [27], [28], [29], [30], [31]. Indeed, a consistent finding between various functional genomic studies in both animal models and humans with obesity and diabetes has been a reduced expression of genes in the BCAA metabolic pathway [for review see 32]. Decreased expression and activity of key regulatory enzymes of BCAA catabolism in liver and adipose tissue in Zucker rats have been reported [33] as well as a marked decrease in hepatic BCAA catabolic enzyme activity models of T2D [34], [35], [36]. Adipose tissue has been suggested to be quantitatively important for whole-body BCAA catabolism [32], [37], [38] and in obesity a consistent finding has been decreased gene expression of BCAA catabolic enzymes in adipose tissue [27], [30], [33], [39]. In contradistinction, some studies, e.g., [21], but not all, e.g., [14], have reported increased Leu oxidation in obesity.

Thus, beyond elevated food intake, it is not clear what mechanisms additionally contribute to the branched chain aminoacidemia of obesity. Therefore, in this study the potential role of BCAA appearance from food intake and proteolysis, along with their rates of disappearance due to protein synthesis and oxidation were determined in obese Zucker fa/fa rats, a genetic model of obesity and insulin resistance. We additionally sought to understand how nutrient signaling to translation initiation in obesity, e.g., [40], could be posited to be elevated on one hand when muscle mass accretion in obese Zucker rats is decreased on the other. To address these questions several approaches were used including metabolomics and determination of whole-body Leu and protein turnover, monitoring [1-^14^C]-α-ketoisocaproate (KIC) using methods outlined by Wolfe and colleagues [41], [42]. Additionally, tissue-specific protein synthesis was measured using [^3^H]-Phe and the flooding dose method. To elucidate the role of changes in the rate-controlling step in BCAA oxidation, total and actual BCKDC activities were also determined. Amino acid and acylcarnitine profiling were also performed to elucidate the status of later steps in BCAA metabolism, incomplete fatty acid oxidation and protein turnover. To the best of our knowledge, acylcarnitines have not been previously characterized in this model. In addition to providing insight into BCAA derived metabolites, other acylcarnitines have been linked to obesity comorbidities such as insulin resistance, alterations in intestinal bacterial flora or have been found to polarize macrophages to the M1 pro-inflammatory phenotype associated with metabolic syndrome [9], [43], [44].

## Materials and Methods

### Ethics Statement

All procedures were conducted after review and approval by the Penn State Hershey Institutional Animal Care and Use Committee (IACUC). The Animal Resource Program, operated by the Department of Comparative Medicine, is accredited by AAALAC International. All animal living conditions are consistent with standards laid forth in the Guide for the Care and Use of Laboratory Animals, 8th edition, published by the National Research Council.

### Animals

Male obese (fa/fa) Zucker and lean control (Fa/?) rats at the age of ∼9 weeks were obtained from Charles River Laboratories (Cambridge, MA, USA). After quarantine they were acclimated to single housing for ∼1 wk while being maintained on a 12∶12 h light dark cycle at ∼21°C and provided free access to water and a standard rodent chow (2018, Harlan Laboratories, Madison, WI). The concentration of protein (18%) and leucine (1.8%) in this diet were supplied by the manufacturer and more than one lot of the diet was used in these studies. Three cohorts of narrow body weight range Zucker rats (n = 10/lean and obese genotype) were used in three separate experiments: (A) Leu metabolism (1-^14^C-Leu), (B) protein synthesis using the [^3^H]-Phe flooding-dose method, and (C) metabolomics/enzyme activity assays. To facilitate comparison of the results from these studies, each of the cohorts experienced similar handling, single housing, infusion (e.g., saline instead of saline with radioisotope), nutritional state and surgical interventions (venous and arterial catheter placement under isoflurane anesthesia) with the exception that animals in the latter cohort were housed for three days prior to catheterization in Nalgene metabolic cages to obtain 24 h urine samples. Although urine was collected daily, only the urine on the third collection day was aliquoted on ice and frozen at −84°C for biochemical analyses. Venous (jugular) and arterial (carotid) catheters were surgically implanted using sterile procedures in all rats under isoflurane anesthesia as previously described [45]. The artery catheter was filled with a viscous solution of heparin (300 units/ml) and 80% polyvinyl pyrolidone (PVP-10; Fisher Scientific, Pittsburgh, PA) to prevent refluxing of blood into the catheter lumen. Rats were kept on a veterinary surgery heating pad for at least two hours after surgery to maintain body temperature and allowed ∼1 week of recovery. Thus, rats were ∼11–12 weeks old at the time of the infusion studies and tissue collection. Only rats with body weights being within 5% of the pre-operative values were used. For body composition analysis, ^1^H-NMR was performed on awake animals for ∼1 minute after body weight determination, as previously described [45]. Blood was collected into heparinized tubes at the times indicated in the methods below.

A goal of these studies was to examine the differences between lean and obese rats as close as possible to the fed state to examine the contributions of various factors in leucine turnover. Thus, 24 h urine measurements and food intake measures were in the fed state. Body weight and compositions were performed in the morning while rats were also in the fed state. However, for other measured endpoints the animals were allowed to eat normally during the dark cycle and then were deprived of food in the morning 2 h before and then during leucine turnover, protein synthesis or tissue sampling. This protocol was used instead of an overnight fasting state, because (a) it decreases the variability of measurement of Leu turnover and protein synthesis in randomly fed rats where the timing of food intake is not controlled, (b) the animals are not normally in the overnight fasted state and (c) an overnight fast can affect both plasma BCAA concentrations and the concentration of enzymes involved in BCAA metabolism [33]. Since the longest of these studies was the Leu infusion study (cohort A), all of the other studies were time-matched the Leu infusion protocol.

At the conclusion of each study animals were anesthetized using isoflurane at which time additional blood was collected for hormone or metabolomic studies. The heparinized blood was maintained on ice until plasma was isolated following centrifugation at 4°C. The plasma was then frozen in liquid nitrogen. At this time tissues were also collected and freeze clamped at the temperature of liquid nitrogen. The tissues were quickly wrapped in labeled foil and kept in liquid nitrogen. Later tissues were powdered under liquid nitrogen. The frozen tissue powders and plasma were transferred to and maintained at −84°C until subsequent biochemistry or analytical studies.

### Amino Acid Analysis and Specific Activity of [1-^14^C]-Leu

Amino acid concentrations were analyzed by two methods that provided different subsets of amino acids and had different sensitivities. The first method measured 10% sulfosalicylic acid (SSA)-extracted amino acids using a BioChrom 30 Amino Acid Analyzer (BioChrom Limited, Cambridge, England) with a cation exchange column with post-column ninhydrin detection at 440 and 570 nm housed in the Hormone Assay and Analytical core of the Vanderbilt Diabetes and Research Training Center. The second method has been previously described [46] and involves pre-column derivatization using Phenomenex EZ-fast reagent. It uses a solid phase sorbent tip to separate derivatized “free” amino acids from proteins and does not involve an acid extraction step; it employs multiple internal standards and an external standard curve for every amino acid. The extracted derivatized amino acids and standards were separated and analyzed by UPLC-MS using a Waters Synapt HDMS hybrid QTOF with Ion Mobility housed in the Penn State College of Medicine Macromolecular Core Facility. Data analysis was completed using the Waters Mass Lynx software.

This UPLC-MS/MS method was employed in order to measure 3-MeHis and resolve it from 1-methyl-histidine (1-MeHis). The distinction is important because the degradation of anserine, from which 1-MeHis arises, and muscle proteins, from which 3-MeHis arises, is thought to occur through different mechanisms. There are two caveats with the assay of these isomers. First, the nomenclature for these two molecules has been reversed in recent years and this change has been adopted by chemical vendors, but not in the biological literature. This has caused confusion [47]. In pilot studies (data not shown), we examined (a) methyl-histidine isomers from two vendors, comparing the chemical structures corresponding to vendor supplied CAS numbers with the structure of the non-myofibrillar, sarcoplasmic dipeptide, anserine, (b) elution time of standards (which in our hands could be resolved with this method) and (c) MS-MS fragmentation/daughter ion profiles. In the pilot studies, new standards obtained from Bachem and Sigma-Aldrich had matching labeling and CAS structures along with unique MS fragmentations and elution times that matched the labeling from each company. However, as expected based on the new nomenclature system, these names were mismatched to anserine’s structure based on biological literature. Therefore, in this paper we used biological nomenclature as opposed to recent chemical nomenclature changes for those structures as suggested by Aranibar et al. [47]. Thus 3-MeHis in their paper refers to the methylated-histidine derived from degradation of muscle myofibrillar fractions whereas 1-MeHis is from the muscle dipeptide, anserine. The pilot studies revealed a second caveat. Methods we received with the Phenomenex EZ-fast reagent kindly provided two recommended MS fragmentation peaks for each methylhistidine isomer. However, for each isomer only one of these two masses tracked with the same pure compound. As a result of these pilot studies and the nomenclature previously suggested for biological studies [47], the daughter ion used for 3-MeHis-Phenomenx reagent adduct that we used was 210 and for 1-MeHis-Phenomenx reagent adduct was 256.

For tissue amino acid analysis, powders of freeze-clamped gastrocnemius and liver were aliquoted (200–300 mg) and then extracted with ice-cold 10% 5-sulfosalicylic acid (SSA, 1∶4, wt/vol) following Polytron homogenization. The homogenates were centrifuged at 3,000 g for 15 min. Plasma was deproteinized with 10% SSA (1∶3, wt/vol) and centrifuged at 8,000 g for 10 min. Aliquots of supernatants were used for amino acid analysis using the above BioChrom 30 Amino Acid Analyzer method. Tissue amino acid concentrations were normalized to the amount of precipitated protein. Protein in the precipitates was measured using the BCA™ protein assay kit (Thermo Scientific, Rockford, IL) after pellets were solubilized with 2.5 ml of 1 N NaOH facilitated by vortexing and heating at 80°C for 30 min.

Leu-^14^C specific radioactivity (SA) was determined as previously described [48]. Briefly, 500 µl aliquots of the supernatants from SSA extracts of plasma, muscle or liver were added to cation exchange columns (1×4 cm) containing Sigma Dowex 50W×8 (200 mesh). The α-keto acids do not bind to this resin and were eluted with 5 ml of 0.01 N HCl. After two deionized water washes (5.0 ml each), Leu and other amino acids were eluted with 8.5 ml of 4.0 N NH_4_OH. One ml of the amino acid eluate was used to determine radioactivity by liquid scintillation counting. Recoveries of [1-^14^C]-Leu added to cold plasma and tissue samples carried through this procedure were ∼98%. Leu ^14^C SA was calculated by dividing ^14^C radioactivity (disintegrations per minute, DPMs) by the amount of Leu present as determined by HPLC.

### Plasma Chemistries, Insulin and Specific Activity of ^14^C-KIC

Plasma glucose and insulin was measured as previously described [49]. Plasma, liver and muscle BCKA concentrations in acidified extracts were measured by HPLC following pre-column O-phenylenediamine (OPD) derivatization of acid deproteinized samples as described [50], with a chloroform extraction for tissues [51]. OPD-derivatized metabolites were separated using a Shimadzu (Columbia, MD) HPLC fitted with a LiChroCART 125-4, Purospher STAR RP-18e (5 µM) analytical column with a LiChroCART 4-4, Purospher STAR RP-18e (5 µM) guard column (Merck, Darmstadt, Germany), which was maintained at 37°C. The chromatograms were analyzed by LC Solutions software (Shimadzu, Columbia, MD). The BCKA and IS peak areas for the standard and plasma samples were quantified, and the concentrations in the plasma samples were calculated based upon a ratio of BCKA area and IS area. Plasma aqueous volume was corrected for protein content with a factor of 0.93.

For specific activity determination, the eluates were collected using a BioRad Model 2110 Fraction Collector (Hercules, CA). The DPMs of ^14^C-radioactivity in the eluted fractions corresponding to KIC was determined using a Beckman 6600IC scintillation counter. Non-radioactive rat plasma was studied after addition of [U-^14^C]-KIC standard and was carried through the above procedure, and a 68% recovery of [U-^14^C]-KIC standard in KIC fractions was used to correct actual ^14^C-KIC radioactivity in plasma samples. Plasma and tissue ^14^C-KIC SA was calculated by dividing radioactivity by KIC concentration in each sample.

### Urinary Creatinine, Carnitine and Acylcarnitine Profiles

Urine creatinine concentration was measured spectrophotometrically using the Jaffe reaction. Urine total and free carnitine, butyrobetaine (the precursor of carnitine) and seventy acylcarnitine species were quantified in the CLIA compliant laboratory of Dr. C.L. Hoppel (Case Western Reserve University, Cleveland, OH) as previously described [52], [53]. The data were expressed in terms of urine creatinine excreted per day.

### [^14^C]-NaHCO_3_ and [1-^14^C]-Leu Infusion Protocol

**Figure 1:**
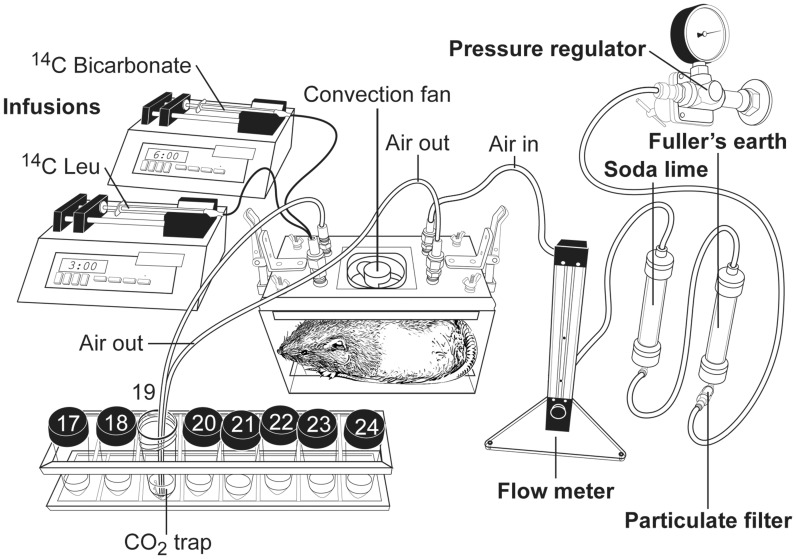
Illustration of experimental set up for measuring Leu flux in rats. After being filtered through mist (Fuller’s Earth) and CO_2_ (Soda lime) absorbent, a constant stream of air flowed through one tube to a closed metabolic cage with a quiet convection fan (Columbus Instruments, Columbus, OH) and out through two outlet tubes which bubbled into a 50 ml conical tube containing Hyamine 10X hydroxide (Perkin Elmer, Waltham, MA)-ethanol (1∶1, vol/vol) for CO_2_ fixation. ^14^CO_2_ samples were collected every 10 min throughout the infusions. The rat was implanted with a jugular vein catheter and infused with [^14^C]-NaHCO_3_ (Moravek Biochemical, Brea, CA) and then [1-^14^C]-Leu (Moravek Biochemical, Brea, CA). [^14^C]-NaHCO_3_ and [1-^14^C]-Leu were each sequentially infused for 2 h. Three blood samples were collected at the 90 and 105 min (0.5 ml) and 120 min (∼3 ml) after start of [1-^14^C]-Leu infusion. A carotid catheter (not shown) came through the same sealed cage port as the jugular cannula pair and was used for blood sampling.

Leu metabolism was measured in conscious rats infused with [1-^14^C]-Leu by monitoring the ^14^CO_2_ in the expired air while the rats rested in a sealed chamber designed for calorimetry studies (Fig. 1). The chamber had constant airflow. Blood was sampled and isotope infused via the arterial and venous catheters, respectively, which entered the cages through silicone sealed ports. Because the ^14^C-CO_2_/bicarbonate pools equilibrate slowly when ^14^C-Leu is infused, pools were rapidly brought into steady-state isotopic equilibrium by first infusing ^14^C-bicarbonate prior to infusion of [1-^14^C]-Leu [41], [42].

At 0900 h, after 2h of food deprivation but free access to water, previously catheterized rats were placed into a closed chamber (Columbus Instruments, Columbus, OH) without food. The chamber as supplied was fitted with a convection fan along with sealed connectors for catheters, air flow-in and -out as shown in Fig. 1. The air stream to the cage was filtered through Fuller’s earth (to trap any oil aerosols) and Soda lime (to adsorb ambient CO_2_) and finely regulated to 1600 ml/min with a Bel-Art Scienceware (#404070125) Aluminum Riteflow Mounted Flowmeter. The air flowed through one rigid inlet tube (Parker Parflex® PE tubing; inch: 0.25 in. O.D. and 0.40 in. wall thickness); air exited the chamber via two outlet tubes of the same diameter to prevent backpressure and flowed into a CO_2_ trap containing 10 ml of Hyamine 10X hydroxide (Perkin Elmer, Waltham, MA)-ethanol (1∶1, vol/vol).

The protocol for the Leu infusion studies was developed from the monograph by Wolfe [42], which includes an initial infusion of labeled bicarbonate to bring the bicarbonate pools into isotopic equilibrium. The initial primed-constant infusion of [^14^C]-NaHCO_3_ facilitates quicker equilibrium of the ^14^CO_2_ pool so that the time for [1-^14^C]-Leu infusion for measuring Leu oxidation can be shorter than infusing [1-^14^C]-Leu alone [41], [42]. The jugular vein catheter was connected to two infusion pumps for sequential infusions of [^14^C]-NaHCO_3_ (Moravek Biochemical, Brea, CA) and [1-^14^C]-Leu (Moravek Biochemical, Brea, CA). Bicarbonate pools were first brought into isotopic equilibrium with [^14^C]-NaHCO_3_ (diluted 50x with sterile 0.9% saline from the original bottle, 59.2 mCi/mmol) at a priming dose of 3.6 µCi (30 µl/min for 3 min) and then continuously infused (0.12 µCi/min, 3 µl/min) for 117 min. [1-^14^C]-Leu (no dilution, 53.8 mCi/mmol) was primed with a dose of 18 µCi (30 µl/min for 3 min and then continuously infused (0.6 µCi/min, 6 µl/min) for 117 min. ^14^CO_2_ samples were collected every 10 min throughout the infusions. The recovery of ^14^CO_2_ of infused [^14^C]-NaHCO_3_ as expired ^14^CO_2_ was determined for CO_2_ fixation efficiency and retention calculation [42]. Three blood samples were then collected from the carotid catheter during the next 90 and 105 min (0.5 ml) and 120 min (∼3 ml) of [1-^14^C]-Leu infusion.

At the conclusion of the infusion protocol, rats were anesthetized with sodium pentobarbital (70 mg/kg) injected into the artery catheter. Indicated tissue samples were quickly collected, freeze-clamped using liquid nitrogen, these samples were quickly foil wrapped and stored at −80°C for biochemical analysis. [^14^C]-NaHCO_3_ samples trapped from expired gas and [1-^14^C]-Leu infusates were diluted 300x with 1 N NaOH and H_2_O, respectively. The diluted infusates and the ^14^CO_2_ Hyamine samples were mixed with 10 ml of Hionic-Fluor (Perkin Elmer, Waltham, MA) and DPMs- in each sample was determined by scintillation counting.

### Calculations of Fluxes of Leu Metabolism

All calculations were carried out at near isotope and substrate steady-state using the formulas below derived by Wolfe and colleagues [41], [42]. Because of the previously described issues of calculating flux from plasma [^14^C]-Leu SA [54], [55], including that [^14^C]-KIC is thought to better represent the intracellular [^14^C]-Leu specific activity, plasma ^14^C-KIC SA was used to calculate Leu turnover and oxidation rates, unless otherwise indicated. The formulas used to were as follows:

#### Leu turnover rate (F)

F (µmol/min/kg) = I (dpm/min)/[SA_KIC_ (dpm/µmol)*wt (kg)], where F is Leu turnover rate (i.e. Leu flux or appearance rate from proteolysis if there was no food absorption); I is [1-^14^C]-Leu infusion rate, SA_KIC_ is plasma [^14^C]-KIC SA; and wt is either body weight or FFM.

#### Leu oxidation rate (Ox)

Ox (µmol/min/kg)  = ^14^CO_2_ (dpm/min)/[SA_KIC_(dpm/µmol)*wt (kg)], where ^14^CO_2_ is the rate of ^14^CO_2_ production corrected by a CO_2_ fixation efficiency and retention factor. This factor was individually determined for each animal. SA_KIC_ is plasma KIC ^14^C SA, and wt is either body weight or FFM as indicated.

#### Rate of proteolysis (PD)

PD (g protein/kg/h) = [F (µmol/min/kg) * 60]/590 (µmol/g protein)], where F is Leu turnover, and 590 represents an average mol of Leu per g of whole-body protein. [42].

#### Rate of protein synthesis (PS)

PS (g protein/kg/h) = [(PD-Ox) * 60]/590.

### Protein Synthesis Using [^3^H]-Phe and Flooding Dose Method

Protein synthesis rates were also determined using the flooding dose approach as previously described [4].

### BCKDC Activity Assay

Branched chain keto acid dehydrogenase complex (BCKDC) extracts from rat tissues were prepared as reported previously [56]. Total and actual activities of the BCKDC in extracts of liver, kidney, gastrocnemius, fat and heart were measured by spectrophotometric assay. The total activity of tissue extracts was measured after full activation of the BCKDC using lambda protein phosphatase [56]. Enzyme activities were also in skeletal muscle, heart and adipose tissue were measured using stable isotope since their activities were too low for the spectrophotometric assay. Briefly, ^13^CO_2_ generated from α-keto[1-^13^C]-isocaproate via BCKDC was measured using an isotope ratio mass spectrometry system [57]. One unit of BCKDC activity refers to the formation of 1 µmol of NADH/min. The activity state of the BCKDC is defined as the percentage of actual (active form) activity/g wet wt relative to total activity (activity of fully activated enzyme)/g wet wt.

### Statistical Analysis

Data are expressed as mean ± SEM, and P ≤ 0.05 was considered as statistically significant. Statistical analysis was performed using Graphpad Prism 6.0 software; two-tailed unpaired *t*-tests were used to compare the difference between the lean and obese groups. To reduce the risk of false positives in HPLC data, we corrected for multiple comparisons using the method of Benjamini and Hochberg as supplied with Graphpad Prism 6.0 software, [58]. The false discovery rate (Q) was set to 1%.

## Results

### Body Compositions, Organ Weights, Plasma Glucose and Insulin Concentrations and 24h-urine Analysis

Each of the three cohorts of rats had similar body weights (Tables 1, 2, 3). At 11 wk of age, the obese Zucker rats already exhibited severe obesity compared with their lean counterparts, based on body weight, body compositions and organ mass (Tables 1, 2, 3 and data not shown). For example, the body weight of obese rats along with their fat mass and fat mass percentage were markedly greater than those in lean controls (Table 1). In contrast, their FFM percentage was less than that in lean controls, although their FFM was greater.

**Table 1:** Body composition of lean and obese rat.

	Lean	Obese	Difference (%)	P Value
Body wt, g	283±3.1	463±7.0	+64	<0.0001
Fat wt, g	60±1.1	174±5.3	+189	<0.0001
Fat, %	21±0.3	37±0.7	+77	<0.0001
FFM, g	178±1.6	221±3.2	+24	<0.0001
FFM, %	63±0.3	48±0.6	−24	<0.0001
Fluid wt, g	19±0.3	35±0.5	+82	<0.0001
Fluid, %	7±0.1	8±0.1	+12	<0.0001

The ^1^H-NMR endpoints were measured in awake fed Zucker rats from cohort A (the cohort used for [^14^C]-Leu metabolism studies), similar results were obtained from cohort B and C (not shown); Values are mean ± SEM; N = 10/group. FFM, fat free mass.

**Table 2:** Body weight, organ weights and plasma glucose and insulin concentrations of lean and obese rats*.

	Lean	Obese	Difference (%)	P value
Body wt	290±9	445±13	+53	<0.0001
Plantaris muscles	0.58±0.04	0.44±0.02	−24	0.008
Gastrocnemius muscles	2.62±0.13	2.08±0.07	−21	0.003
Soleus muscles	0.30±0.02	0.26±0.01	NS	0.1
Epididymal fat pads	3.0±0.26	11.5±0.45	+283	<0.0001
Perirenal fat pads	1.5±0.10	6.8±0.90	+353	<0.0001
Liver	7.8±0.46	16±0.8*	+105	<0.0001
Kidneys	2.3±0.16	2.8±0.19	+22	0.05
Heart	0.80±0.03	0.88±0.02	+10	0.05
Plasma glucose, mg/dl	160±6	173±8	NS	0.21
Plasma insulin, ng/ml	0.24±0.05	10.9±1.7	+4442	<0.0001

* Weights in g were obtained from cohort B (the cohort used for protein synthesis using the [^3^H]-Phe) and represent the sum of the weight of the left and right side where applicable. Plasma values are from cohort A (the cohort used for [^14^C]-Leu metabolism studies). Values are means ± SE, n = 9–10 per group. Percent differences were determined before rounding. NS indicates not significant (P>0.05). Data on amount of protein per g wet weight for some tissues is shown in Table 7.

**Table 3:** Food, fluid, protein and Leu intakes with urine outputs in lean and obese Zucker rats*.

	Lean	Obese	Difference (%)	P value
Body weight, g	280±3.4	455±4.7	+63	<0.0001
Food intake, g/d	19±0.9	37±0.9	+98	<0.0001
Food intake, g/100 g body wt	6.7±0.3	8.2±0.2	+23	0.0006
Protein intake, g/d	3.5±0.17	6.9±0.17	+97	<0.0001
Protein intake, g/100 g body wt	1.2±0.05	1.5±0.04	+23	0.0006
Leu intake, g/d	0.34±0.02	0.67±0.02	+97	<0.0001
Leu intake, g/100 g body wt	0.12±0.005	0.15±0.004	+23	0.0006
Water intake, mL/d	22±0.9	45±5.9	+105	0.0008
Water intake, mL/100 g body wt	8±0.33	10±1.3	NS	0.14
24-hour urine volume, mL	10.0±0.5	29.0±3.1	+190	<0.0001
Urine vol, mL/100 g body wt	3.6+0.2	6.4±0.7	+78	0.001
Urine [Creatinine], mM	8.7±0.5	3.4±0.3	−61	<0.0001
24-hour urine creatinine, mg	9.4±0.3	10.3±0.5	NS	0.13

* Data from cohort C (the cohort in which no radioactivity was used for metabolomics/enzyme activity assays). Values are means ± SE, n = 9–10 per group. NS indicates not significant (P>0.05). Leucine and protein intake are determined from the values provided by the manufacturer of the diet. Small differences in percent changes (+98 vs +97%) are due to removal of significant figures and rounding.

Body composition changes were also apparent in wet weights of tissues, exemplified by those from cohort B (Table 2). Body weight of obese animals in this cohort were ∼50% greater than lean controls. While obese soleus weights were not statistically different, the mass of the plantaris and gastrocnemius muscle masses were ∼20% lower than those from lean animals. Given the overall much larger body size of the obese Zucker rats, all three muscles were disproportionately smaller in obese compared to lean rats. However, whether this difference in muscle weight reflects a failure to grow, an atrophic response, or a combination of these is unclear and was not further addressed. Conversely, weights of epididymal and perirenal fat pads were increased ∼4-fold, and the weight of the liver was increased more than 2-fold. Finally, the weight of the heart and kidney was increased 10–20% in obese rats.

As anticipated, the obese rats consumed more food, protein and Leu per day along with more water (Table 3). However while body weights in cohort C were 63% greater, food, protein and Leu intakes were approximately double that of lean rats. Hence, even when normalized for body weight, obese rats consumed ∼23% more food, Leu and protein (Table 3).

To decrease the variability of plasma measures and other metabolic assessments, overnight food deprivation is frequently used. However as mentioned in the Methods, we have found that this duration of food deprivation alters plasma BCAAs and enzymes in lean and obese animals [33]. Moreover this is not the normal state for either lean or obese rats. As a compromise, we allowed the animals to eat normally during the dark cycle and then food deprived them for 2 h in the morning before the leucine turnover studies. As shown in Table 2, this design resulted in no significant differences in the plasma glucose concentrations between the lean and obese animals with low variability, less than 5%. However, these values were elevated compared to published and unpublished overnight food deprived glucose values data not shown and earlier references, e.g., [49], [59]. In contrast, plasma insulin was elevated approximately 45 fold by obesity (Table 2) demonstrating severe insulin resistance expected from this model.

Twenty-four hour urine collections were made after the animals had a 48 h acclimation period in the metabolic cages. Consistent with their increased body weight (Table 3), obese rats of the same cohort had a 190% increase in their 24-h urinary excretion (Table 3). There was no significant difference in the amount of creatinine excreted per day; however the greater volume of urine formed in the obese rats resulted in urinary creatinine dilution (Table 3).

### Metabolic Profiles Focusing on BCAAs and their Metabolism

**Figure 2:**
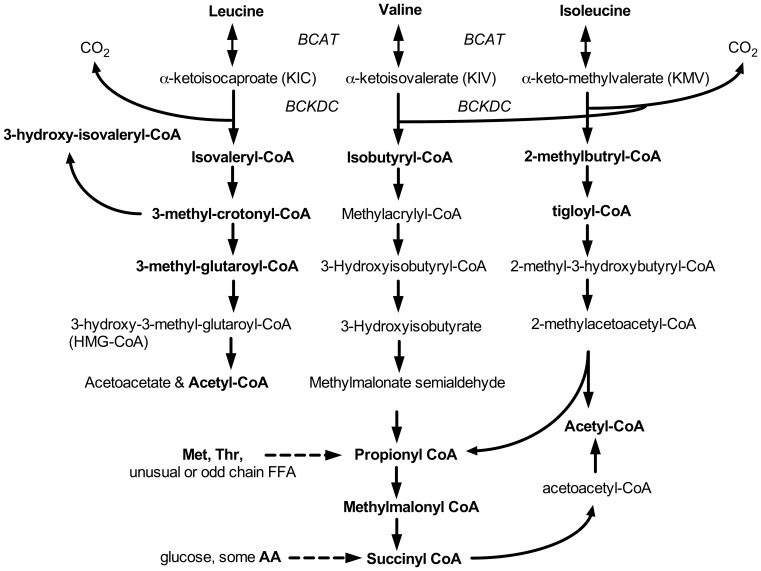
Schematic of whole-body BCAA metabolism. Ketoacids are formed by reversible transamination catalyzed by the mitochondrial or cytosolic isoforms of branched chain amino acid transaminase (*BCAT*). The action of the branched chain keto acid dehydrogenase complex (*BCKDC*) in the mitochondrial matrix leads to the evolution of CO_2_ from the 1-carbon of the keto acids including KIC, which was ^14^C labeled and measured from the expired air in these studies. Subsequent intramitochondrial metabolism leads to the formation of various acyl-coenzyme A (R-CoA) esters that can reversibly form acylcarnitines (not displayed). Neither FAD and NAD Cofactors nor CO_2_ and H_2_O substrates are displayed. Bold font indicates metabolites or corresponding acylcarnitines that were detected and measured quantitatively in the 24 h urines (Table 4–5). AA, amino acids.

**Figure 3:**
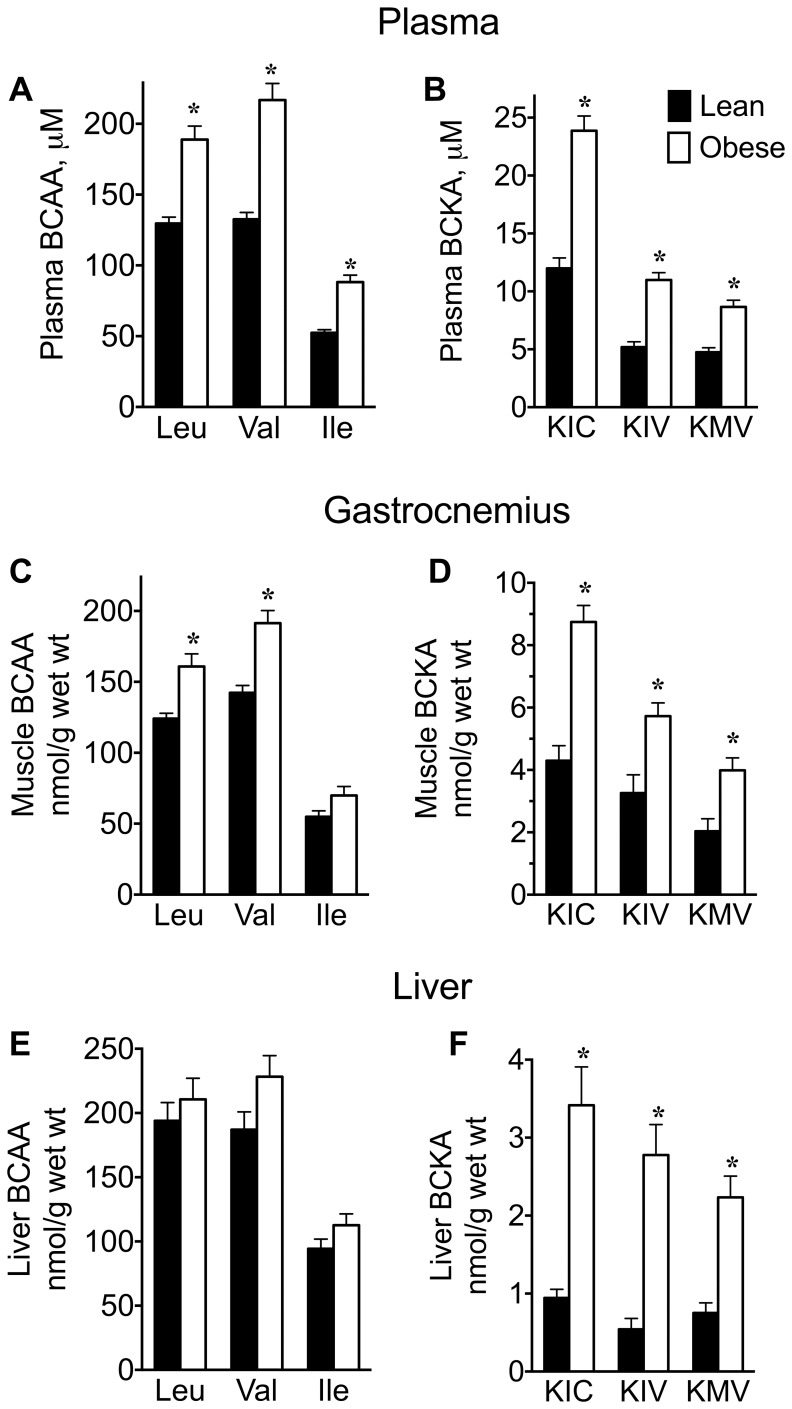
Plasma and tissue BCAA and BCKA concentrations in lean and obese Zucker rats. Concentrations of BCAAs (Leu, Val and Ile: A, C, E) and BCKAs (KIC, KIV, and KMV: B, D, F) are shown for plasma (A, B), gastrocnemius muscle (C, D) and liver (E,F). Bars are mean ± SE; *P<0.05, n = 9 and 8 determinations for lean and obese rats, respectively.

For reference the metabolic pathway for the branched chain amino acids and their ketoacids is shown in Fig. 2. Targeted assays for metabolites in this pathway and metabolomics were performed on plasma, tissues and urine, as appropriate (Fig. 3, Tables 4, 5, 6). Monitoring plasma amino acids and acylcarnitines provides a snapshot of metabolites at a single point in time since both plasma amino acids and acylcarnitines can change dynamically within minutes [60], [61]. Conversely, excretion of metabolites in the urine collected over 24 h provides a more integrated picture of whole body metabolism over the course of a full day. Therefore, we also measured amino acids and acylcarnitines concentrations in 24 h urine samples normalized to the 24 h creatinine concentration. As BCAAs and their metabolites were a primary focus, they are discussed first.

**Table 4:** 24 h Urine amino acids in lean and obese Zucker rats (µmol/g creatinine).

	Lean	Obese	Difference (%)	p-value
3-MeHis	168±15.5	476±44.7	+183	<0.0001
1-MetHis	7.78±0.80	9.11±1.06	NS	0.31
α-Aminobutyric acid	5.30±0.62	11.85±1.77	+123	0.003
Alanine	255±30.1	554±71.5	+118	0.001
Arginine	35.0±3.45	50.3±5.48	NS	0.03
Asparagine	130±13.7	235±19.9	+81	0.0004
Aspartate	103±19.7	193±24.6	NS	0.01
ß-Aminoisobutyric acid	13.4±0.71	16.4±1.24	NS	0.04
Cystine	11.4±0.71	27.4±2.56	+140	<0.0001
Citrulline	8.93±0.62	17.9±1.59	+100	<0.0001
4-aminobutyric acid (GABA)	56.2±4.07	88.3±7.25	+57	0.001
Glutamine	172±15.7	356±43.7	+107	<0.0001
Glutamate	577±26.3	544±72.7	NS	0.668
Glycine	1353±269	2909±237	+115	0.0004
Glycine-proline (dipeptide)	68.4±3.18	91.9±8.13	NS	0.02
Histidine	144±5.30	210±12.6	+46	<0.0001
δ-Hydroxylysine	8.04±1.15	15.1±3.18	NS	0.06
4-Hydroxyproline	0.53±0.09	4.60±0.97	+767	0.0005
Isoleucine	30.7±3.27	70.2±11.9	+129	0.004
Leucine	34.3±3.89	91.1±12.1	+166	0.0003
Lysine	431±33.2	640±49.5	+48	0.003
Methionine	96.4±9.64	190±26.5	+97	0.004
Ornithine	60.2±6.72	100.8±15.2	NS	0.02
Phenylalanine	39.8±1.77	82.0±5.13	+106	<0.0001
Proline	187±8.84	422±26.1	+126	<0.0001
Sarcosine	82.1±11.2	149±8.04	+81	0.0002
Serine	73.5±4.60	115±9.90	+56	0.001
Threonine	81.2±5.75	191.0±19.9	+135	<0.0001
Tryptophan	13.9±1.68	19.3±3.45	NS	0.178
Tyrosine	53.9±3.98	66.7±8.22	NS	0.18
Valine	48.1±4.42	119±14.9	+146	0.0002

Values are means ± SEM; n = 10 rats per group. NS indicates a P>0.05 or that the comparison was flagged as a potential false discovery when the Q was set to 0.01 [58].

**Table 5:** Urine acylcarnitines in lean and obese Zucker rats (µmol carnitine/g creatinine).

Analyte	Lean	Obese	%	P-value	Analyte	Lean	Obese	%	P-value
Sum, Total & Free Carnitine(s)	166±18.4	399±28.5	+141	<0.0001	S-3-Hydroxy-decanoylcarnitine	0.025±0.001	0.061±0.004	+149	<0.0001
Free carnitine	108±19.3	298±22.8	+177	<0.0001	5-Decynoylcarnitine	0.139±0.008	0.122±0.008	NS	0.154
Total - Free	58±2.7	101±8.0	+73	<0.0001	Lauroylcarnitine	0.014±0.001	0.036±0.003	+158	<0.0001
Sum of [Acylcarnitines]	20±1.6	59±6.3	+199	<0.0001	Trans-2-dodecenoylcarnitine	0.005±0.001	0.012±0.003	NS	0.02
Butyrobetaine	41±3.7	105±7.7	+155	<0.0001	R-3-Hydroxy-lauroylcarnitine	0.003±0.000	0.009±0.001	+156	0.0009
Acetylcarnitine	4.2±0.52	11.2±0.97	+163	<0.0001	S-3-Hydroxy-lauroylcarnitine	0.007±0.001	0.019±0.003	+170	0.003
Propionylcarnitine	0.24±0.02	0.60±0.04	+154	<0.0001	Myristoylcarnitine	0.009±0.001	0.022±0.002	+155	<0.0001
Butyrylcarnitine	0.11±0.006	0.31±0.029	+180	<0.0001	Myristoleoylcarnitine	0.024±0.002	0.053±0.006	+118	0.0002
Isobutyrylcarnitine	0.22±0.041	2.16±0.226	+874	<0.0001	Cis-5-tetradecenoylcarnitine	0.012±0.002	0.023±0.004	NS	0.04
R-3-Hydroxy-butyrylcarnitine	0.039±0.003	0.105±0.007	+172	<0.0001	Trans-2-tetradecenoylcarnitine	0.020±0.001	0.046±0.004	+127	<0.0001
S-3-Hydroxy-butyrylcarnitine	0.038±0.002	0.104±0.009	+170	<0.0001	Cis,cis-5,8-tetradecadienoylcarnitine	0.020±0.001	0.050±0.004	+157	<0.0001
Unidentified hydroxy-C4-carnitine	2.74±0.49	25.84±4.06	+842	<0.0001	R-3-Hydroxymyristoylcarnitine	0.007±0.000	0.017±0.001	+151	<0.0001
Valerylcarnitine	0.015±0.001	0.022±0.003	NS	0.053	S-3-Hydroxy-myristoylcarnitine	–	–		–
Isovalerylcarnitine	0.072±0.006	0.263±0.023	+266	<0.0001	Hydroxy-C14∶1	0.007±0.001	0.019±0.003	+164	0.002
3-Hydroxy-isovalerylcarnitine	0.56±0.10	2.6±0.29	+367	<0.0001	Palmitoylcarnitine	0.009±0.002	0.024±0.005	+154	0.009
2-Methyl-butyrylcarnitine	0.15±0.02	0.52±0.06	+240	<0.0001	Palmitoleoylcarnitine	0.009±0.001	0.017±0.002	+88	0.004
Pivaloylcarnitine	0.078±0.004	0.195±0.009	+151	<0.0001	Trans-2-hexadecenoylcarnitine	0.012±0.001	0.024±0.002	+107	<0.0001
Tigloylcarnitine	0.052±0.009	0.26±0.023	+412	<0.0001	R-3-Hydroxy-palmitoylcarnitine	0.018±0.001	0.047±0.005	+161	<0.0001
3-Methyl-crotonylcarnitine	0.010±0.001	0.029±0.004	+185	0.00025	S-3-Hydroxy-palmitoylcarnitine	0.005±0.000	0.012±0.001	+126	0.0001
Hexanoylcarnitine	0.063±0.005	0.11±0.007	+78	<0.0001	Hydroxy-C16∶1	0.005±0.000	0.013±0.001	+172	<0.0001
R-3-Hydroxy-hexanoylcarnitine	0.010±0.001	0.026±0.003	+165	<0.0001	Stearoylcarnitine	0.011±0.001	0.022±0.002	+106	0.0004
S-3-Hydroxy-hexanoylcarnitine	0.012±0.001	0.032±0.003	+164	<0.0001	Linoleoylcarnitine	0.014±0.001	0.036±0.003	+158	<0.0001
Phenylacetylcarnitine	0.80±0.21	1.18±0.32	NS	0.330	α–Linolenoylcarnitine	0.010±0.001	0.015±0.002	NS	0.06
Phenylpropionylcarnitine	0.012±0.001	0.031±0.002	+150	<0.0001	γ-Linolenoylcarnitine	0.024±0.001	0.048±0.004	+100	<0.0001
4-phenyl-butyrylcarnitine	0.005±0.000	0.013±0.001	+147	<0.0001	R-3-Hydroxy-stearoylcarnitine	0.012±0.001	0.030±0.003	+164	<0.0001
Benzoylcarnitine	0.034±0.003	0.067±0.007	+98	0.0003	S-3-Hydroxy-stearoylcarnitine	0.002±0.000	0.004±0.001	+154	0.001
4-Methyl-hexanoylcarnitine	0.019±0.001	0.050±0.004	+163	<0.0001	Hydroxy-C18∶1	0.001±0.000	0.003±0.000	+93	0.002
Octanoylcarnitine	0.040±0.002	0.082±0.005	+102	<0.0001	Hydroxy-C18∶2	0.001±0.000	0.003±0.000	+100	0.0006
R-3-Hydroxy-octanoylcarnitine	0.12±0.017	0.24±0.031	+95	0.0045	Hydroxy-C18∶3	0.003±0.000	0.004±0.000	NS	0.03
S-3-Hydroxy-octanoylcarnitine	0.020±0.002	0.042±0.005	+113	0.001	Malonylcarnitine	3.0±0.11	3.3±0.16	NS	0.11
Valproylcarnitine	0.026±0.002	0.073±0.006	+185	<0.0001	Succinylcarnitine	2.1±0.06	2.5±0.22	NS	0.062
Cis-3,4-Methylene-heptanoylcarnitine	0.10±0.011	0.21±0.017	+106	<0.0001	Methyl-malonylcarnitine	2.6±0.13	3.2±0.27	NS	0.054
4-Methyl-octanoylcarnitine	0.021±0.001	0.049±0.003	+140	<0.0001	Ethyl-malonylcarnitine	0.099±0.005	0.103±0.014	NS	0.81
2,6-Dimethyl-heptanoylcarnitine	0.021±0.001	0.050±0.004	+141	<0.0001	Glutaroylcarnitine	0.30±0.009	0.37±0.040	NS	0.11
Decanoylcarnitine	0.012±0.001	0.033±0.002	+174	<0.0001	Adipoylcarnitine	0.39±0.018	0.59±0.057	+49	0.005
Cis-4-Decenoylcarnitine	0.028±0.002	0.049±0.003	+79	<0.0001	3-Methyl-glutaroylcarnitine	0.16±0.009	0.22±0.025	NS	0.03
Cis-3, 4-Methylene-nonanoylcarnitine	0.26±0.014	0.22±0.013	NS	0.08	Suberoylcarnitine	0.22±0.015	0.36±0.029	+65	0.0005
R-3-Hydroxy-decanoylcarnitine	0.016±0.001	0.040±0.002	+155	<0.0001					

Values are means ± SEM; n = 10 rats per group, % refers to percent difference Obese/Lean. NS indicates P>0.05 or that comparison was flagged as a potential false positive when the Q was set to 0.01 [58]. HMG-carnitine, oleoylcarnitine and S-3-hydroxy-myristoylcarnitine that are part of the profile were not detected.

**Table 6:** Plasma concentrations (µM) of amino acids and related metabolites in lean and obese rats.

	Lean	Obese	Difference (%)	P Value
Taurine	85±9.3	131±12.5	+54	0.008
Urea	3941±26.1	5151±463	NS	0.02
Aspartic Acid	6.6±0.3	7.6±0.4	NS	0.061
Threonine	167±8.8	164±10.7	NS	0.8
Serine	168±8.1	142±6.3	NS	0.02
Asparagine	56±1.9	45±2.6	−21	0.002
Glutamate	57±1.8	110±4.8	+94	<0.0001
Glutamine	709±17.9	438±17.3	−38	<0.0001
Glycine	247±12.2	112±4.2	−55	<0.0001
Alanine	323±29.4	455±21.6	+41	0.002
Citrulline	72±5.4	62±3.3	NS	0.14
Valine	133±4.8	217±11.8	+64	<0.0001
Cysteine	70±2.1	62±2.8	NS	0.04
Methionine	37±1.8	32±2.6	NS	0.09
Isoleucine	52±2.2	88±5.0	+68	<0.0001
Leucine	130±4.4	189±9.5	+46	<0.0001
Tyrosine	49±2.0	52±2.2	NS	0.28
Phenylalanine	75±2.9	77±2.1	NS	0.60
Ornithine	35±1.9	37.5±5.7	NS	0.68
Lysine	388±21	313±20	NS	0.02
1-Methylhistidine	13±1.6	7.6±0.4	−43	0.0024
Histidine	72±2.4	60±1.9	−17	0.001
Tryptophan	71±2.6	61±6.0	NS	0.13
Arginine	122±6	87±7.5	−29	0.002
Proline	146±23	128±11	NS	0.50

Values are means ± SEM; n = 10 rats per group. NS indicates a P>0.05 or that the comparison was flagged as a potential false discovery when the Q was set to 0.01 [58].

Plasma concentrations of Leu, Val and Ile (determined with the amino acid analyzer) were increased 46–68% in obese rats compared to lean rats (Fig. 3A). Plasma concentrations of the corresponding α-ketoacids of Leu, Val and Ile (α-ketoisocaproate (KIC), α-ketoisovalerate (KIV), and α-keto-β-methylvalerate (KMV), respectively) were increased 87–112% in obese rats (Fig. 3B). Gastrocnemius BCAA concentrations, expressed as per g wet tissue, were increased ∼20% (Fig. 3C), whereas skeletal muscle BCKAs were increased 76–103% in obese rats (Fig. 3D). Hepatic BCAA concentrations were unaltered (Fig. 3E), whereas KIC, KIV and KMV concentrations were elevated 261%, 413% and 197%, respectively, in livers from obese rats (Fig. 3F).

**Figure 4:**
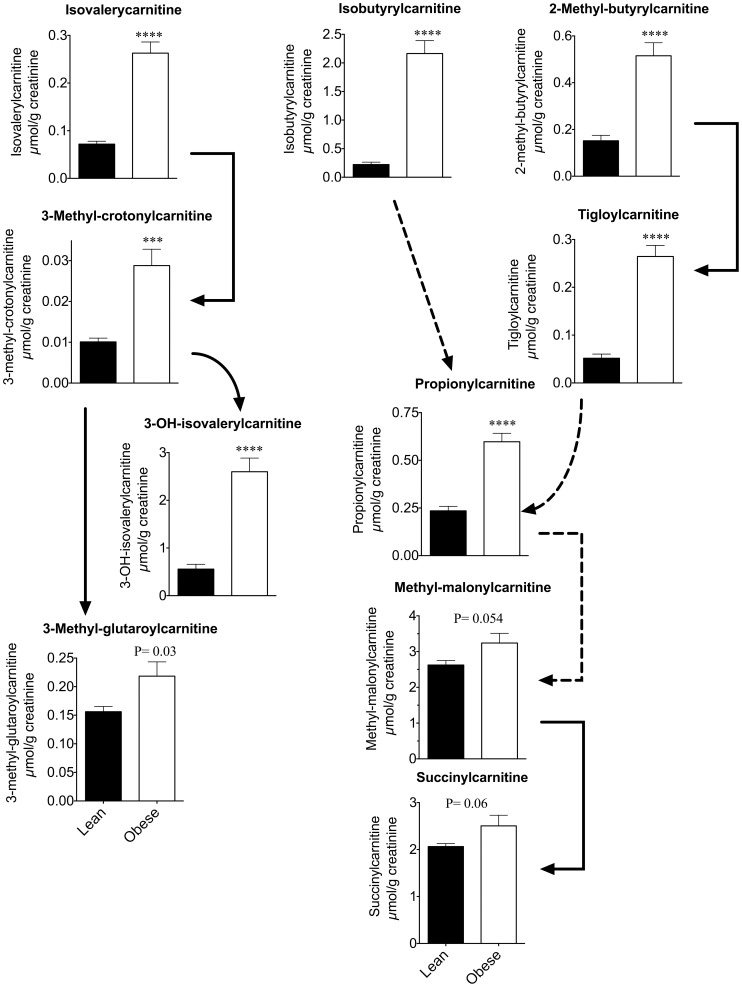
24-h urine acylcarnitines derived from BCAA metabolism. The graphs are organized to align with the pathway in Fig. 2. A solid arrow indicates a single enzymatic step between the metabolism of the corresponding Co-A species from which the acylcarnitines are derived; a broken arrow indicates several steps. Mean ± SE are shown, *** p<0.001, **** p<0.0001.

Urinary concentrations of BCAAs were increased in obese rats by 130–170% (Table 4). During the metabolism of BCAAs, various acyl Coenzyme-A (CoA) esters are formed (Fig. 2). Within the mitochondria, carnitine helps maintain the acyl-CoA to free Co-A ratios by reversibly exchanging the acyl groups to form acylcarnitine esters. The acylcarnitines so formed can be exported from the mitochondria and appear in the plasma and urine. Urinary concentrations of acylcarnitines corresponding to the acyl-CoA products of BCKDC were all markedly elevated in the 24-h urine samples of obese rats when corrected for creatinine, and a number of their downstream metabolites were also elevated or showed a trend for increase (Fig. 4, Table 5). In the pathway of Leu metabolism, obesity increased urinary concentrations of isovalerylcarnitine (266%), 3-methyl-crotonylcarnitine (185%) and 3-hydroxy-isovalerylcarnitine (367%). There was also a trend for increase in urine 3-methyl-glutaroylcarnitine. The carnitine ester of the next product in the pathway, HMG-CoA, is not normally detectable in urine and was not detected here. In the pathway of Val metabolism, urinary isobutyrylcarnitine was elevated 874% in the obese rats. Other unique carnitine esters that are formed from Val metabolism were not detected, whereas metabolites formed from Ile metabolism were present in the urine. In the Ile metabolism pathway, the carnitine derivative formed from the product of BCKDC, 2-methyl-butyrylcarnitine, was increased 240% in obese rats. Propionyl-carnitine was also elevated 154% in obese animals compared to lean. There was a trend for an obesity-induced increase in urinary methymalonylcarnitine (p = 0.054) and succinylcarnitine (p = 0.06, Fig. 4), but the magnitude of these changes was modest compared to upstream metabolites.

### Whole Body Leu Turnover and Protein Metabolism

**Figure 5:**
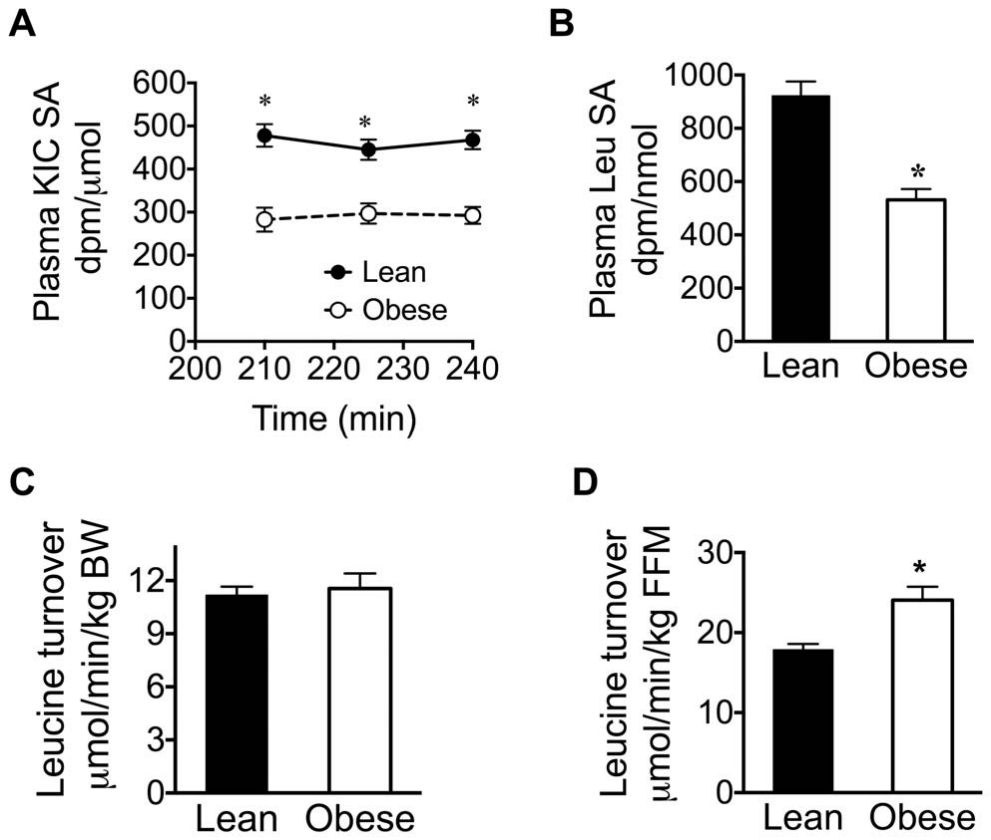
Plasma ^14^C KIC and Leu specific activity (SA) and turnover in lean and obese Zucker rats. Plasma ^14^C-SA for KIC (A) and Leu (B) were calculated dividing DPM radioactivity of KIC and Leu with their amounts in each sample. Leu turnover (Leu flux or rate of appearance) was calculated by dividing Leu infusion rate with the plasma ^14^C KIC SA and expressed per kg of body wt (BW) (C) or fat free mass (FFM) (D). Values are mean ± SE; * P<0.05, n = 9 and 8 for lean and obese rats, respectively.

The elevations in the plasma and urine BCAAs and their metabolites in the obese rats were ∼2–8 fold higher than the ∼20% increased Leu intake corrected for body weight suggesting that changes in BCAA metabolism also occur. Leu turnover studies using [1-^14^C]-Leu [41], [42] were performed to evaluate other arms of BCAA metabolism that might be altered and that could explain the obesity-related changes in BCAAs and lower muscle mass despite aminoacidemia. Fig. 5A shows that during the infusion of [1-^14^C]-Leu the plasma ^14^C-KIC SA within each group did not differ at three time points during the last hr of the infusion indicating a steady-state metabolism of infused ^14^C-Leu (Fig. 5A). However, the average plasma ^14^C-KIC SA was 59% lower in obese than lean rats, and plasma ^14^C-Leu SA after 120 min of ^14^C Leu infusion was 42% lower in obese rats (Fig. 5B). These results are consistent with the markedly increased Leu plasma concentration in obese rats. Using plasma ^14^C-KIC SA, the calculated whole-body Leu turnover rate normalized to body weight did not differ between the two groups of rats (Fig. 5C). However, when the denominator was expressed in terms of kg FFM, Leu turnover was 35% greater in obese rats (Fig. 5D). Since ^14^C-Leu and -KIC SAs were similarly altered in lean and obese rats, the change in Leu turnover calculated with ^14^C-Leu SA was not different from that calculated based on ^14^C-KIC SA. The absolute Leu turnover rates using the ^14^C-Leu SA were lower in both groups than the corresponding rates calculated from ^14^C-KIC SA (data not shown), in agreement with a previous report [62].

**Figure 6:**
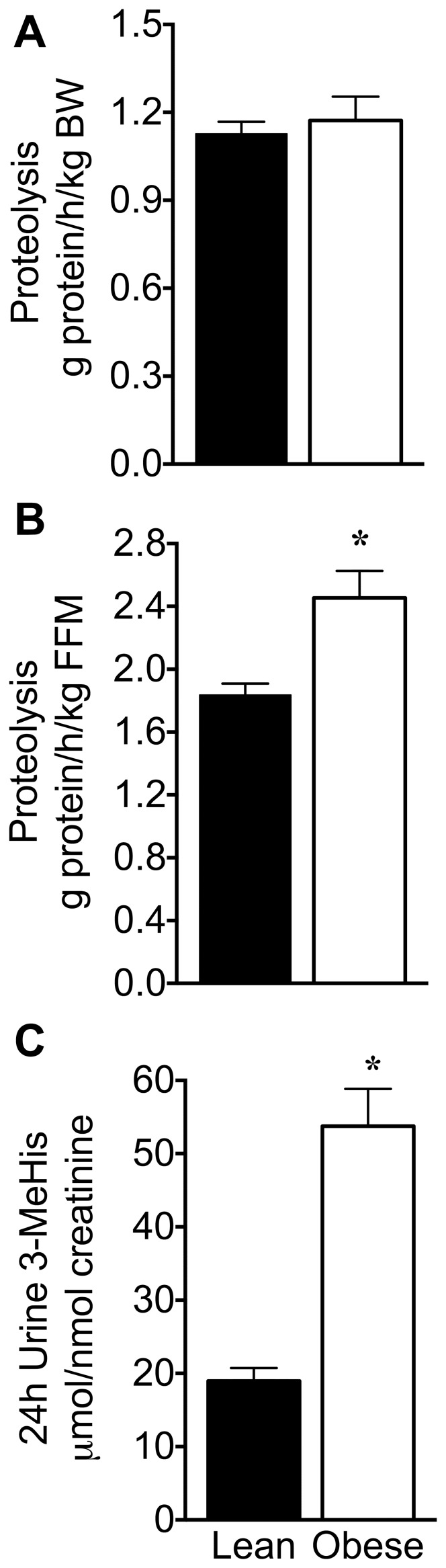
Whole-body proteolysis and muscle proteolysis in lean and obese Zucker rats. Whole-body proteolysis was expressed either per kg of body weight (BW) (A) or fat free mass (FFM) (B). Values are mean ± SE; * P<0.05, n = 9 and 8 for lean and obese rats, respectively, in A and B. The ratio of urinary muscle derived 3-MeHis to creatinine (C) was calculated from 24-h urinary amounts of 3-MeHis and creatinine to normalize 3-MeHis excretion by creatinine (D). Values are mean ± SE; * P<0.05, n = 10 for both groups in C and D.

As observed with Leu turnover, the rate of whole-body proteolysis in the postabsorptive state was also unaltered when normalized with body weight; however, it was 35% greater in obese than lean animals when normalized with FFM (Fig. 6A &B). There was other evidence of increased protein tissue breakdown from the 24-h urine data. The ratio of 3-MeHis to creatinine was increased 183% obese Zucker rats compared to lean controls, suggesting elevated proteolysis of muscle and possibly other tissues (e.g., gut) in the obese animals (Fig. 6C and Table 4). In contrast, 1-MeHis, the amino acid from the muscle dipeptide anserine was unchanged in urine and lower in the plasma (Table 4). These findings suggest a specific effect on proteolysis as opposed to muscle toxicity where both methylhistidines isomers increase in urine [47]. 4-Hydroxyproline is a modified amino acid found in collagen. During collagen degradation, 4-hydroxyproline is not reused and is thus excreted in the urine similar to 3-MeHis; it is considered a marker of collagen turnover [63]. Urine concentrations of 4-hydroxyproline were elevated 766% in obese Zucker rats (Table 4). Notably, the urinary concentrations of 4-hydroxyproline and 3-MeHis demonstrate the largest increases in obese rats compared to other amino acids.

**Figure 7:**
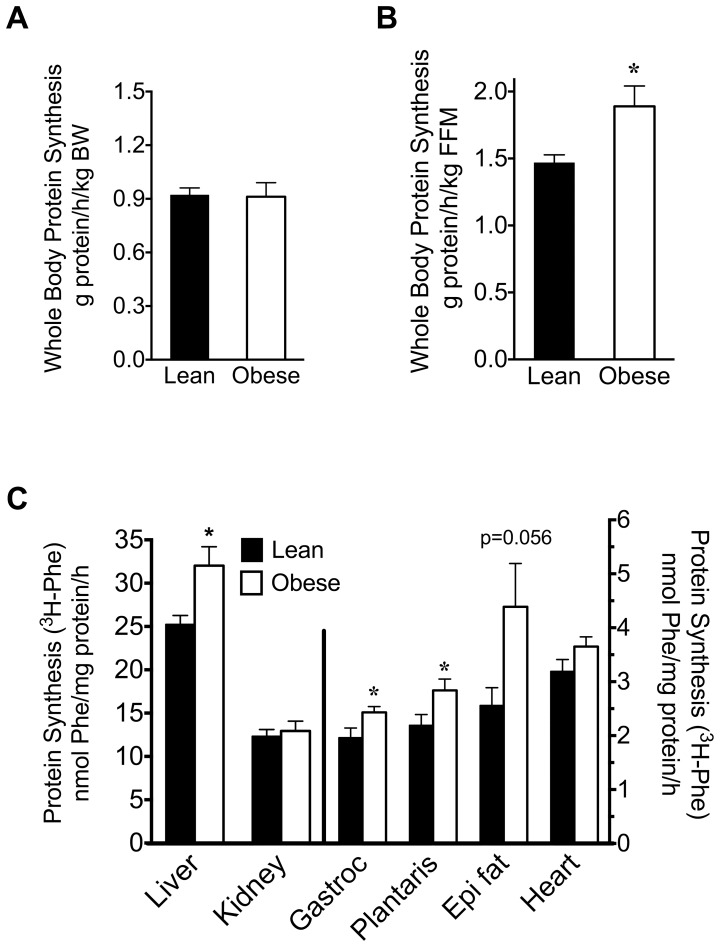
Protein synthesis in whole-body and selected tissues of lean and obese Zucker rats. Whole-body protein synthesis (A,B) was expressed either per kg of body weight (BW, panel A) or fat free mass (FFM, panel B). *P<0.05, n = 9 and 8 for lean and obese rats, respectively. (C) In vivo protein synthesis in indicated tissues was measure using ^3^H-phenylalanine (^3^H-Phe) flooding dose method. Values are mean ± SE; *P<0.05, n = 10 for both groups in panel C.

Rates of whole-body protein synthesis per kg body weight were unaltered in obese rats, but were increased 29% compared to lean animals when the values were normalized to FFM (Fig. 7A & B). Collectively, these data suggest that both lean mass protein synthesis and degradation are elevated in obese Zucker rats.

### Protein Synthesis Using ^3^H-Phe and the Flooding dose Method

We used the flooding-dose approach to measure protein synthesis in different tissues (Fig. 7C). Protein synthesis rates in liver, gastrocnemius and plantaris were increased ∼25–30% in obese Zucker rats compared to lean controls (Fig. 7C). Adipose tissue protein synthesis in the obese rats was variable compared to the leans, with a trend toward an increase. Conversely, no significant change in protein synthesis was detected in heart or kidney from obese rats.

### Whole-body Leu Oxidation and Tissue BCKDC Activity

**Figure 8:**
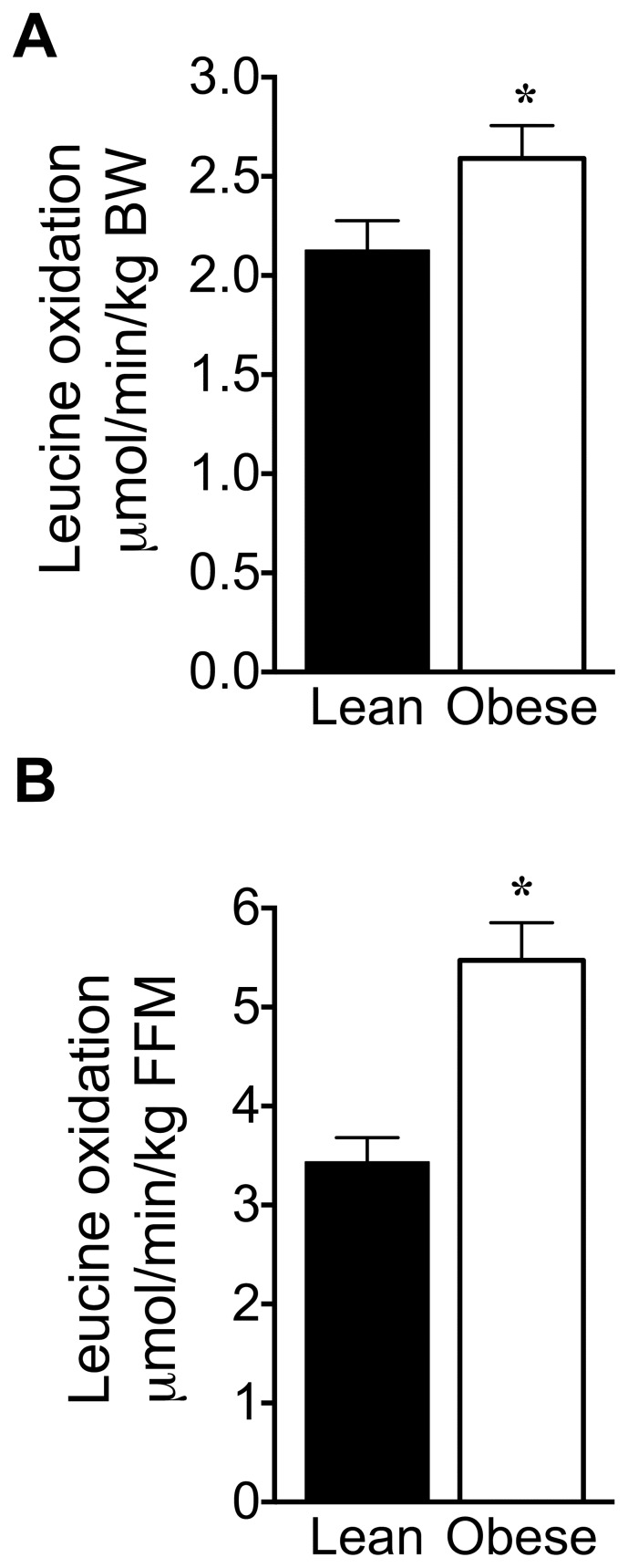
Whole-body leucine oxidation in lean and obese Zucker rats. Whole-body leucine oxidation was expressed either per kg of body weight (BW) (A) or FFM (B). Leu oxidation was calculated by dividing the rate of ^14^CO_2_ production with plasma ^14^C-KIC SA. Values are mean ± SE; * P<0.05, n = 9 and 8 for lean and obese rats.

The rate of whole body Leu oxidation was increased in obese rats regardless of whether data were normalized to body weight (+22%) or FFM (+60%, Fig. 8A & B). Since ^14^CO_2_ generated during the infusion of [1-^14^C]-Leu is derived from BCKDC-catalyzed oxidative decarboxylation, our Leu oxidation data reflect an elevated whole-body BCKDC-catalyzed activity in obese rats. Increased BCAA oxidation in obesity seems inconsistent with our previous findings of decreased content, activity and phosphorylation-mediated inactivation of key regulatory enzymes of BCAA catabolism in liver and adipose tissue of obese and T2D animal models [13], [33], [34], [35], [36]. Therefore we measured enzyme activity of BCKDC, the rate-limiting enzyme of BCAA catabolism (Table 7).

**Table 7:** Branched chain keto acid dehydrogenase activity in lean and obese Zucker rats*.

	Lean	Obese	%	P-value
**Liver**				
BCKDC Actual Activity, U/g wet wt	0.68±0.04	0.30±0.03	−55	<0.0001
BCKDC Total Activity, U/g wet wt	1.46±0.05	0.93±0.04	−36	<0.0001
% Active BCKDC	46.4±1.5	32.0±2.4	−31	<0.0001
Protein (mg)/g wet wt	222±6	211±5	NS	0.18
Citrate Synthase, µmol/min/g tissue	8.53±0.5	6.94±0.24	−19	0.01
BCKDC Actual Activity, U/whole liver	5.28±0.28	4.77±0.40	NS	0.31
Citrate Synthase Activity, µmol/min/whole liver	66.5±4.2	111±3.9	+67	<0.0001
**Kidney**				
BCKDC Actual Activity, U/g wet wt	0.53±0.04	0.28±0.02	−47	<0.0001
BCKDC Total Activity, U/g wet wt	1.17±0.05	0.66±0.07	−44	<0.0001
% Active BCKDC	40.6±3.0	44.3±2.4	NS	0.14
Protein (mg)/g wet wt	180±3	159±6	−12	0.006
Citrate Synthase Activity, µmol/min/g tissue	41±3.0	40±2.3	NS	0.79
BCKDC Actual Activity, U/total kidney mass	1.21±0.08	0.77±0.07	−36	0.0006
Citrate Synthase Activity, µmol/min/total kidney mass	93.0±7	112±7	NS	0.071
**Heart**				
BCKDC Actual Activity, U/g wet wt	0.41±0.034	0.15±0.016	−66	<0.0001
BCKDC Total Activity, U/g wet wt	1.34±0.04	1.15±0.04	−14	0.003
% Active BCKDC	31±2	12±2	−61	<0.0001
Protein (mg)/g wet wt	176±10	169±4	NS	0.52
Citrate Synthase Activity, µmol/min/g tissue	99±2	105±1	NS	.11
BCKDC Actual Activity, U/whole heart	0.33±0.03	0.13±0.02	−61	<0.0001
Citrate Synthase Activity, µmol/min/whole heart	79±2	92±1	+17	<0.0001
**Gastrocnemius Muscle** a,				
BCKDC Actual Activity, nmol/min/g wet weight tissue	1.7±0.41	0.7±0.12	−59	0.03
Protein (mg)/g wet wt	136±7	169±7	+24	.0024
Citrate Synthase Activity, µmol/min/g tissue	19±.5	25±1	+28	0.0002
BCKDC Actual Activity, nmol/min/both muscles	4.3±1.1	1.4±0.3	−67	0.016
Citrate Synthase Activity, µmol/min/both muscles	50±1	51±2	NS	0.79
**Epididymal Fat** a				
Citrate Synthase Activity, µmol/min/g tissue	0.41±0.019	0.21±0.021	−49	<0.0001
Citrate Synthase Activity, µmol/min/fat pad	1.2±0.06	2.5±0.24	+104	<0.0001
Protein (mg)/g wet wt	177±29	57±6.9	−68	0.0005

* Data from cohort C. Enzyme activities were measured at 30°C. Values are means ± SEM; n = 10 rats per group. NS indicates not significant, P>0.05.

a Muscle and adipose tissue BCKDC activities were measured using the ^13^C method [57], which does not use U indicating µmol of NADH formed. BCKDC total activities were unavailable for muscle; neither total nor actual BCKDC activities were available for adipose tissue. Unfortunately, activation of BCKDH using lambda phosphatase did not work in these experiments. Additionally, the adipose tissue values were very low and too close to the level of detection and contamination from room air and are not reported.

Compared to other tissues, liver had the highest BCKDC activity in lean rats. Total and actual BCKDC activity in liver was decreased by 54% and 68%, respectively, in obese Zucker rats compared to lean controls. For comparison purposes, hepatic citrate synthase activity, measured as a mitochondrial marker, decreased only 19%. Thus, a portion of the decreased BCKDC activity is attributable to decreased hepatic mitochondrial content in obese rats. As the body composition of the obese rats was different, we also calculated enzyme activities per organ. When hepatic BCKDC activity is considered in this context, that is with liver mass as the denominator, the actual BCKDC activity did not differ between the lean and obese animals. Citrate synthase activity per liver increased 65% supporting the idea that the enlarged liver also has more mitochondria, but this change was not proportional to the increase in the liver mass, which was increased 107% in obesity (Table 7). Renal BCKDC actual and total activities per g wet weight were decreased by approximately half in obese rats with no change in BCKDC activity state or citrate synthase. Actual renal BCKDC per activity per kidney was still lower (Table 7). While cardiac citrate synthase activity per g wet weight increased 6% in obese rats, BCKDC total and actual activity declined 14% and 66%, respectively, with a 61% decrease in activity state. There was a 66% decrease in actual BCKDC activity per heart.

Muscle and adipose tissue BCKDC activities were too low for the spectrometric assay. Therefore a ^13^C methodology was used and this necessitated that the BCKDC activity be expressed in different units. Unfortunately, in this experiment the lamda phosphatase activation of the enzyme did not work so only actual BCKDC activities are available. In addition, the adipose tissue activities were too close to the level of detection and contamination of room air CO_2_, so they are not provided. Actual activity measurements were therefore only available for muscle (Table 7). Actual gastrocnemius BCKDC activity was lower independent of the normalization denominator (Table 7). In contrast, citrate synthesis activity per muscle was unchanged and actual BCKDC activity per muscle was decreased 67%.

Adipose tissue citrate synthase activity decreased in obese rats per g wet weight, but citrate synthase per fat pad increased about 2-fold in obesity due to the increase in fat pad size.

Understanding how obesity changes the tissues in terms of protein content per g wet wt is also important to facilitate comparison to previous Western blot studies which were loaded by mg of protein, e.g. [33]. Along with changes in the masses of the tissues, Table 7 shows that obesity is associated with changes in the ratio of protein to mg wet wt. Notably, this ratio was markedly decreased (68%) in adipose tissue from obese rats. The ratio was unchanged by obesity in the liver and heart, decreased in kidney, and increased in gastrocnemius from obese rats.

### Other Metabolic Findings

Plasma and tissue concentrations of non-BCAA amino acids and amino acid-related metabolites were significantly altered in obese rats (Table 6 and 8). For example, compared to lean controls, plasma glutamate and alanine were increased 94 and 41%, respectively, whereas plasma glutamine was decreased 38%. As for the other gluconeogenic amino acids, plasma threonine and serine concentrations were unaltered, but plasma glycine decreased 55% in obese rats. While the plasma urea concentration tended to be increased (P = 0.017), urea cycle intermediates ornithine and citrulline were unaltered and plasma concentrations of arginine were lower (−31%) in obese rats. Plasma taurine was increased 54%, however, the other sulfur-containing amino acids (methionine and cysteine) were unchanged in obese rats. There were no differences in aromatic amino acids (phenylalanine, tyrosine, and tryptophan) between the obese and lean rats. Liver phenylalanine and tyrosine concentrations were unaltered; however, muscle concentrations of phenylalanine, tyrosine, and methionine were decreased 24%, 31% and 51%, respectively (Table 8).

**Table 8:** Muscle and liver concentrations (nmol/mg protein) of BCAA, methionine, and two aromatic amino acids in lean and obese Zucker rats^a^.

		Lean	Obese	Difference (%)	P Value
Gastrocnemius	Valine	1.48±0.05	1.80±0.09*	21.3	0.005
	Leucine	1.29±0.03	1.51±0.08*	17.1	0.02
	Isoleucine	0.57±0.04	0.65±0.06	NS	0.28
	Methionine	0.49±0.03	0.24±0.01*	−50.7	<0.0001
	Tyrosine	0.90±0.03	0.63±0.05*	−30.6	0.0002
	Phenylalanine	0.79±0.03	0.60±0.03*	−23.8	0.0003
Liver	Valine	1.75±0.12	2.45±0.16*	40.3	0.0025
	Leucine	1.82±0.13	2.26±0.16*	24.0	0.046
	Isoleucine	0.88±0.07	1.20±0.09*	35.2	0.01
	Methionine	0.23±0.02	0.29±0.01*	26.1	0.02
	Tyrosine	0.57±0.05	0.65±0.04	NS	0.23
	Phenylalanine	0.65±0.06	0.77±0.05	NS	0.15

Data from Cohort A. Values are means ± SEM (expressed per mg protein not g wet as in Fig. 3); n = 9–10 rats per group. NS indicates not significant the P is greater than 0.05.

There was a general hyperaminoacidemia in the urine when corrected for g of creatinine (Table 4). BCAAs, 4-hydroxyproline, 3-MeHis and cystine showed the largest changes. Notably, the 24-h urinary excretion of some amino acids (serine, asparagine, glutamine, glycine, lysine, and histidine) was elevated in obese Zucker rats in contrast to plasma. Urinary excretion of glutamate was unaltered despite being the most elevated amino acid in plasma. However, a related metabolite, urinary butyrylcarnitine, which can be derived from α-ketoglutarate and glutamate metabolism [64], was elevated (Table 4).

The urinary concentrations of free carnitine, its precursor butyrobetaine and a number of acylcarnitines were significantly elevated in urine from obese Zucker rats (Table 5). The proportion of free to total acylcarnitine was reduced from 54% in lean rats to 33% free in obese animals, further implying an increase in acylcarnitine formation in obesity and consistent with sum of the measured acylcarnitines shown in Table 5. A general increase in acylcarnitines and 3-OH acylcarnitines derived from fatty acid oxidation was apparent in obesity, including those of intermediate chain length, suggesting a greater prevalence of incomplete long-chain fatty acid β-oxidation in the obese rats (e.g., see highly significant increases in decanoylcarnitine and its related metabolites including the S- and R-isomers of 3-hydroxy-decanoylcarnitine). Urinary pivaloylcarnitine, an isobaric isomer of isovalerylcarnitine derived from so-called “neo fatty acids” (fatty acids with a terminal tertiary butyl group), was also elevated in obesity (Table 5).

Acylcarnitine metabolites derived from BCAA oxidation were among the greatest changes in obesity, as described earlier (Fig. 4). Demonstrating this specificity, while isovalerylcarnitine and 3-hydroxy-isovalerylcarnitine were elevated 266 and 367%, valerylcarnitine was not changed. However, another highly elevated species was a hydroxylated C4-acylcarnitine (842%) for which a standard was not available during this analysis. This unknown peak is not R-3-hydroxy-butyrylcarnitine or S-3-hydroxy-butyrylcarnitine (a.k.a. beta-hydroxy-butyrylcarnitine) which were elevated ∼170% in urine from obese rats. Cis-3, 4-methylene-nonanoylcarnitine, an acylcarnitine believed to be derived from catabolism of a parent fatty acid emanating from gut bacteria [65], was not significantly changed in obese rats; however, cis-3,4-methylene-heptanoylcarnitine was increased (Table 5).

## Discussion

Obesity-associated rises in systemic BCAAs and their metabolites were observed in plasma, tissues and 24-h urine of the Zucker rats. While BCAAs were elevated in plasma and muscle, the percent increase in the BCKAs was higher suggesting that BCKAs rather than branched-chain aminoacidemia might be a more sensitive metabolic signal of obesity. Factors that could potentially contribute to modifying BCAAs/BCKAs in obesity include BCAA intake, BCAA oxidation in addition to protein synthesis and degradation. Our findings suggest that all of these processes are altered in the obese Zucker rat. However, only BCAA intake (higher), actual and total tissue BCKDC activity (lower) and protein degradation per FFM (higher) changed in a direction that would positively contribute to elevating circulating BCAAs and BCKAs in obesity. Opposing these rises in BCAAs, whole body protein synthesis per FFM was also increased, however, tissue specific changes were restricted to a few tissues. The effect on protein synthesis may be due in part to elevated nutrient signaling arising from the branched chain aminoacidemia. BCKDC activity decreased in all tissues and previous studies indicate it should decline even further in adipose tissue, for review see [32], though we were unable to measure it herein for technical reasons. These adaptations to obesity should help preserve BCAAs and BCKAs; however, obese rats nevertheless showed increased Leu oxidation.

### Role of Food Intake and BCAA Oxidation in Elevating BCAAs in Obesity

An obvious factor contributing to elevated BCAAs and BCKAs in the obese rats appears to be increased BCAA intake, because animals were sampled in the fed to early-postabsorptive state. The mitochondrial isoform of branched chain amino acid transaminase (BCATm) catalyzes the first step in BCAA metabolism in most peripheral tissues. However this enzyme is absent in liver thereby allowing BCAAs in particular to rise postprandially so they may act as a circulating signal of a protein-containing meal. We found that BCAA intake per g body weight was 23% greater in obese animals. While the percent rise in food intake is not as great as changes in BCKAs or metabolites in various compartments, food intake is clearly a contributing factor. It is noteworthy that virtually all other studies examining blood BCAA in obese humans or rodents were conducted in the overnight-fasted state, suggesting that obesity-associated increases in blood BCAA in those cases could not be due solely to differences in dietary input.

Another potential factor that can contribute to elevated BCAAs in obesity is their rate of disappearance via complete BCAA oxidation, which could potentially be reduced in the obese state. Our observation of increased urinary BCAA-derived metabolites in obese Zucker rats is consistent with increased overall BCAA catabolism, at least through the first steps of BCAA oxidation. Consistent with this view, whole-body Leu oxidation as measured by 1-^14^C-Leu radiotracer was also increased, with the caveat that evolved ^14^CO_2_ only marks the first step in Leu oxidation and does not imply complete oxidation. A decrease in the concentration/activity of enzymes downstream in the BCAA metabolic pathway relative to the incoming substrate load might contribute to accumulation of upstream metabolites reflective of incomplete BCAA oxidation. An example of this is our finding of elevated urinary 3-OH-isovalerycarnitine in obese rats. Elevation of this urine metabolite is used clinically to signal a block or deficiency in 3-methylcrotonyl-CoA carboxylase. Consistent with this possibility, genomic studies indicate down-regulation of gene expression along the whole BCAA metabolic pathway in liver and/or adipose tissue in the insulin-resistant or obese state, for reviews see [13], [32], [33]. The issue of complete versus incomplete BCAA oxidation, the relative roles of substrate excess versus enzyme activities *in situ*, and the tissue origins of systemic and urinary BCAA derivatives are areas that warrant further study.

Our oxidation measurements indicated that Leu oxidation was increased in obese rats consistent with some previous clinical studies, e.g. [14]. This increase occurred despite declines in the actual activity of the enzymatic step responsible for the ^14^CO_2_ evolution in all the tissues studied. When calculations were performed using organ mass as the denominator, a different picture emerged. That is actual BCKDC activity is still decreased in kidney, heart and muscle, but the whole liver BCKDC actual activity is not changed due to obesity.

We could not measure BCKDC activity in adipose tissue due to very low activity and in other previous studies in vitro activities from adipose tissue have also been low compared to other tissues [37]. However, our previous study showed that BCATm and BCKDC are expressed in adipose tissue at levels comparable to other tissues [66]. In agreement with those findings, Herman et al. [32] reported that valine oxidation in adipose tissue explants was 6-fold greater than in extensor digitorum longus muscle and that adipose tissue transplant lowered BCAA concentrations in BCATm KO mice. These data suggest that adipose tissue is a quantitatively important site of whole body BCAA metabolism and imply that adipose tissue lysates contain an inhibitor to which the enzyme is not normally exposed to within mitochondria in vivo (e.g., FFA or FFA-soaps etc., in the lysates). This possibility not withstanding, as there was no obesity-induced change in adipose tissue BCKDC phosphorylation in our previous study [33], we calculated the amount of BCKDC E1-α subunit per fat pad correcting for changes in adipose tissue protein/g wet weight ratio. Here again, BCKDC activity per fat pad is still lower in obesity (data not shown).

Thus, we speculate that increased whole-body BCAA oxidation in obesity may be the result of a substrate effect in liver (a key tissue for BCKA catabolism) based on two observations: (1) the amount of actual hepatic activity per animal (i.e. per liver) in obese animals does not change compared to lean animals and (2) the substrate for hepatic BCKDC, BCKAs, were elevated to a greater extent in the liver of obese animals (∼3-fold) than in either muscle or plasma. While we were surprised that the concentrations of BCAAs per gm of liver were not different, this may also be due to changes in body composition, that is a dilution of hepatic BCAAs due to the more than doubling of the liver size. Acute elevations in BCKAs normally activate BCKDC [67], however, while BCAAs and BCKAs were elevated in several tissues of obese Zucker rats, the percent of active enzyme was lower in a number of tissues. Although the reason for this is not known, we posit that BCAA oxidation have been further elevated by obesity if the observed adaptations in BCKDC activity had not occur.

We also found decreased BCKDC activity in the heart. Previous studies have shown that a regulatory defect in BCKDC due to loss of the BCKDC phosphatase (PP2Cm) leads to a partial reduction in BCKDC activity and elevations of plasma BCKAs and BCAAs [68] leading to an intermittent form of Maple Syrup Urine Disease [68], [69], [70]. While whole body leucine oxidation is increased in obese Zucker rats, in a number of tissues there appears to be a loss of metabolic capacity together with elevations in tissue and plasma BCAA and BCKD plasma concentrations that are comparable to those observed in the PP2Cm knock out (KO) mouse. Loss of PP2Cm due to a mutation in human PP2Cm or PP2Cm KO results in cells with elevated ROS production and stress kinase activation [69], [70], which in turn has been linked to cardiac dysfunction [69]. Since cardiac BCKDC activity is impaired in obese Zucker rats, further studies are needed to determine the potential contribution of defective BCKA metabolism in obesity to cardiovascular dysfunction associated with obesity and whether such effects may extend to other obesity co-morbidities associated with elevated ROS and stress kinase activation.

### Role of Protein Turnover in Elevating BCAAs in Obesity and Lean Mass Changes

Two other factors potentially underlying the elevations in BCAAs in obesity are protein synthesis and protein degradation. Hyperaminoacidemia and elevated plasma Leu have been linked to increased mTOR activity [10], [22], [23] and PKC pathway activation [71], [72]. These pathways have been linked to activation of protein synthesis consistent with our observations in obese rats. However, these are several caveats with this idea. First we did not measure these signaling pathways in the present study. Another issue is that the relationship between hyperaminoacidemia and protein synthesis in other models has been reported to be transient when amino acids or Leu are elevated [73], [74]. Insulin may also be involved in the activation of protein synthesis, but it also difficult to assess the role of insulin in these changes since both the plasma insulin and tissue insulin resistance is greatly increased in this model. A limitation of is that we did not determine if the lean and obese animals were in equivalent absorptive states at the time of these measurements. Therefore, while we did observe increased whole-body protein synthesis per FFM in the obese rats, the mechanism underlying these changes needs to be further investigated. Nevertheless, the data are consistent with the tissue-specific increase in protein synthesis in liver, muscle and possibly adipose tissue. However, such changes would not contribute, but rather blunt the obesity-associated rise in circulating and tissue BCAA and BCKA concentrations.

The increase in the size of liver and fat in this model is consistent with elevated rates of protein synthesis. This increased muscle protein synthesis in obese rats is contrary to our data showing that muscle mass is decreased or unchanged in obesity and therefore suggests a possible compensatory role for protein degradation. Indeed, whole-body proteolysis was elevated when normalized to lean body mass. Those findings are consistent with previous studies in Zucker rats in the food deprived, postabsorbtive state finding increase protein degradation relative to protein synthesis [24], [25], [26]. Increased proteolysis was also evident from the marked increase in urinary 3-MeHis, in agreement with Chan et al. [24] and 4-OH-proline from obese rats. As 3-MeHis originates from skeletal as well as other muscles including smooth muscle, we cannot exclude the possibility that some of the obesity-induced increase in 3-MeHis is from increases in the size of the gastrointestinal tract. Similarly, the skin mass is likely greater and this could be a source of 4-OH-proline. Thus, changes in body composition may in part explain why increases in these amino acids were greater than the estimate of FFM proteolysis. The mathematical difference between the whole body protein synthesis and degradation in the leucine flux studies is not statistically significant so it is unclear whether there are disproportionate changes in protein synthesis vs degradation corrected for FFM. Nevertheless, protein synthesis and degradation were elevated along with elevated urine 3-MeHis and 4-hydroxyproline and this supports the idea of increase net daily protein turnover consistent with more growth and larger body mass in Zucker rats. The increased pool size related to this elevated turnover likely supports the elevations in BCAAs in obesity while increased protein synthesis in some tissues, increased excretion and increased BCAA oxidation acts to blunt those elevations.

It is noteworthy that hyperaminoacidemia in lean animals is usually associated with increased protein synthesis and decreased protein degradation, e.g. [22]. In contradistinction, hyperaminoacidemia in obese Zucker rats was associated with an increase in both protein synthesis and degradation. If aminoacidemia underlies the elevated protein synthesis in obese Zucker rats, which remains to be established, these data suggest that the normal mechanisms which decrease protein degradation in lean animals in response to branched chain aminoacidemia are either not operating or being overridden by a more dominant catabolic signal. Currently, the nature of this putative signal is unclear.

A limitation of our current study is that we did not measure protein turnover in individual tissues as would be possible by measuring arterial-venous differences. Hence using our approaches we did not assess the relative role of each tissue to proteolysis, which may be differ owing to the decreased demand for physical activity and smaller muscle sizes in the obese animals along with the larger liver.

The finding, that obese Zucker rats simultaneously increased the rate of two opposing and energy-consuming processes, protein synthesis (based on lean body mass) and protein degradation, suggests a futile cycle for amino acids similar to that in obese rats for glucose, pyruvate and triglyceride [75], [76], [77]. Activating futile cycles would seem to promote energy wasting, not obesity. For example, similar to Zucker rats, BCATm KO mice eat more food and have elevated protein synthesis and urinary 3-MeHis [78]. However, BCATm KOs are lean and they resist obesity caused by high fat feeding. This phenotype is associated with increased energy expenditure attributed to futile protein-amino acid cycling. There are several possible explanations for why Zucker rats are obese despite an increase in protein synthesis and degradation. In contrast to BCATm KO mice, Zucker rats have other obesity-promoting changes including greater food intake, reduced physical activity [79] and compromised thyroid status [80], [81]. Thyroid hormone promotes inefficiency of energy utilization through multiple mechanisms and thereby increases heat production, which is reduced in Zucker rats [79] but increased in BCATm KO mice [78]. These and perhaps other energy sparing factors in the Zucker rat presumably override the increased energy needs caused by increased protein turnover and other futile cycles.

### Metabolomic Findings

In our study plasma alanine increased however paradoxically plasma glycine decreased. Increased plasma alanine and decreased glycine was also reported by Felig et al. [8] in human obesity, although increased circulating alanine is associated with catabolic states, serving as an important carrier of amino acids from muscle to liver and can arise from transamination of glucose-derived pyruvate carrier increased muscle catabolism and can arise from transamination of pyruvate derived from glycolysis [82], [83], [84], [85]. However, Ruderman and Berger [84] found that alanine and glycine efflux from the muscle rose in concert in both starvation and diabetes. As with Felig et al. [8] in human obesity, the underlying mechanism for the decrease in plasma glycine in these insulin resistant obese Zucker rats is also not clear.

A number of urinary acylcarnitines and 3-OH acylcarnitines were elevated by obesity including acylcarnitines of intermediate chain length (exemplified by decanoylcarnitine and the S- and R-isomers of 3-hydroxy-decanoylcarnitine) that may be indicative of increased and incomplete β-oxidation [86] in rodents [87], [88] and humans, reviewed in [89]. The latter may be the result of elevated FFA tissue availability associated with insulin resistance in obese rats [59].

Our data show that isobutyrylcarnitine (metabolite of Ile) is elevated 874% in obesity, more than any other metabolite including the BCAAs. Despite the magnitude of this elevation, other unique downstream metabolites of Ile were not detected in lean or obese animals, in contrast to a number of metabolites found for Leu and Val. Increased isobutyrylcarnitine along with propionylcarnitine were previously identified in insulin-resistant obese human plasma [9]. In contrast, in obese weight-matched women, those subjects with T2DM displayed reduced plasma propionylcarnitine concurrent with worsening blood glucose control [64], raising the possibility that the dynamics of amino acid catabolism to propionyl-CoA is context-specific [90].

We observed an ∼800% increase in an unidentified C4-OH acylcarnitine. One possibility is that this C4-OH-acylcarnitine is derived from 2-hydroxy-butyrate a metabolite whose blood concentration correlates with insulin resistance and glucose intolerance [13], [28], [91]. Urinary pivaloylcarnitine, a neo fatty acid and isomer of isovalerylcarnitine, was also elevated. It is unclear whether the neo fatty acid precursors of this acylcarnitine originate from soy in the vegetable protein rodent chow or metabolism of gastrointestinal flora.

A urine amino acid arising from collagen, 4-OH-proline, was greatly elevated in obese Zucker rats and has not been previously linked to obesity. However, it is recognized to be elevated in obese individuals during food restriction [92], and in obesity, it is thought to represent increased turnover/remodeling of extracellular matrix in adipose or lean tissues rather other than bone [93]. Further studies are needed to determine whether these metabolites may be useful urine markers for glucose intolerance, insulin resistance and/or obesity risk.

### Conclusions

In conclusion, we report that BCKAs were elevated more than BCAAs in plasma and tissues of obese rats. Thus, BCKAs may represent a more sensitive metabolic signature compared to BCAAs for obesity or insulin resistance. In future studies, following BCKAs concentrations and metabolic capacity may be important for several reasons. As mentioned already, circulating BCKAs are thought to be better representatives of the intracellular pool of BCAAs [42]. In addition, in studies on glial and neuronal cells with defective BCKDC, BCKAs were more toxic than BCAAs [94]. Thus, concerning recent conflicting data around whether elevating BCAAs are beneficial or detrimental for health, it may be valuable to bring measures of BCKAs and their metabolism into the discussion. Whether the circulating BCAA elevations arise from the diet in the context of normal or impaired tissue metabolism leading to BCKA accumulation may be important in this regard.

Our results also show that the elevated plasma BCAAs in obese Zucker rats arise in part from increased BCAA intake, a decrease in the actual activity of BCKDC in peripheral tissues and increased protein degradation. Excess BCAAs appeared to increase protein synthesis and BCAA oxidation; these processes probably contribute to dampening the rise in systemic BCAA concentrations. Elevated leucine and protein turnover also appear to contribute. Alterations in protein turnover could underlie the disproportionately smaller muscles in these rats as previously suggested by Chan et al [24]. A surprising observation was that while BCKDC activity in liver and other tissues was decreased, consistent with our previous studies in both non-diabetic and diabetic Zucker rats [34], [35], [36], whole body Leu oxidation increased in the obese rats consistent with the finding of obesity-related elevations in many acylcarnitines derived from BCAA metabolism. We speculate that increased BCKDC-mediated Leu oxidation in this model of obesity is a substrate effect owing both to BCKAs being elevated to a greater extent in liver compared to muscle and plasma, along with unchanged actual BCKDC activity when the denominator is per liver instead of g wet weight. A caveat is that while the Zucker rat has a very strong and multifaceted obesity phenotype, not all of the changes observed in this model may be generalizable to all human obesities. Further studies are needed to determine which of these pathways and changes observed in this model, beyond the elevation in BCAAs, are also observed in humans.
